# Co-receptor and co-stimulation blockade for mixed chimerism and tolerance without myelosuppressive conditioning

**DOI:** 10.1186/1471-2172-7-9

**Published:** 2006-04-25

**Authors:** Luis Graca, Stephen Daley, Paul J Fairchild, Stephen P Cobbold, Herman Waldmann

**Affiliations:** 1 Sir William Dunn School of Pathology, University of Oxford, Oxford OX1 3RE, UK; 2 Instituto de Medicina Molecular, Faculdade de Medicina, Universidade de Lisboa, P1649-028 Lisbon, Portugal; 3 Instituto Gulbenkian de Ciência, P2780-156 Oeiras, Portugal

## Abstract

**Background:**

A major challenge in the application of marrow transplantation as a route to immunological tolerance of a transplanted organ is to achieve hematopoietic stem cell (HSC) engraftment with minimal myelosuppressive treatments.

**Results:**

We here describe a combined antibody protocol which can achieve long-term engraftment with clinically relevant doses of MHC-mismatched bone marrow, without the need for myelosuppressive drugs. Although not universally applicable in all strains, we achieved reliable engraftment in permissive strains with a two-stage strategy: involving first, treatment with anti-CD8 and anti-CD4 in advance of transplantation; and second, treatment with antibodies targeting CD4, CD8 and CD40L (CD154) at the time of marrow transplantation. Long-term mixed chimerism through co-receptor and co-stimulation blockade facilitated tolerance to donor-type skin grafts, without any evidence of donor-antigen driven regulatory T cells.

**Conclusion:**

We conclude that antibodies targeting co-receptor and co-stimulatory molecules synergise to enable mixed hematopoietic chimerism and central tolerance, showing that neither cytoreductive conditioning nor 'megadoses' of donor bone marrow are required for donor HSC to engraft in permissive strains.

## Background

Bone marrow transplantation (BMT) has widespread therapeutic potential in the treatment of hematological malignancies, genetic defects in the hematopoietic system and autoimmunity [1-4]. The goal of achieving solid organ transplantation tolerance may also be facilitated by the induction of mixed hematopoietic chimerism following the transplantation of donor bone marrow (BM) [5]. It has been a long held assumption that the engraftment of bone marrow transplants requires the creation of "space" in the host. "Space" has usually been created using myeloablative conditioning regimens that include gamma irradiation or cytotoxic agents, both associated with undesirable side effects. As a consequence, there have been efforts to minimize the myeloablative conditioning by developing protocols where engraftment is facilitated with the combination of monoclonal antibodies (mAbs) [6]. The intensity of myelosuppressive conditioning strategies, such as low-dose total body irradiation or busulfan, is able to be reduced by the use of anti-CD40L mAbs ("co-stimulation blockade") [7-9]. Large or "mega-doses" of donor BM have also been shown to be helpful in promoting engraftment [10]. "Mega-doses" of donor bone marrow and anti-CD40L mAbs in the presence [11,12] or absence of donor specific transfusion [13,14], are sufficient to ensure stable bone marrow engraftment in the absence of myelosuppression. We now demonstrate that a combination of mAbs (targeting CD4, CD8 and CD40L), previously shown capable of inducing dominant transplantation tolerance to allogeneic skin grafts [15], permits the engraftment of donor marrow in some strain combinations. This leads to long-term chimerism and transplantation tolerance without the need for mega-doses of donor bone marrow or myelosuppressive conditioning.

## Results

### Mixed chimerism and tolerance using a conventional dose of donor BM without the need for myelosuppression

**Figure 1:**
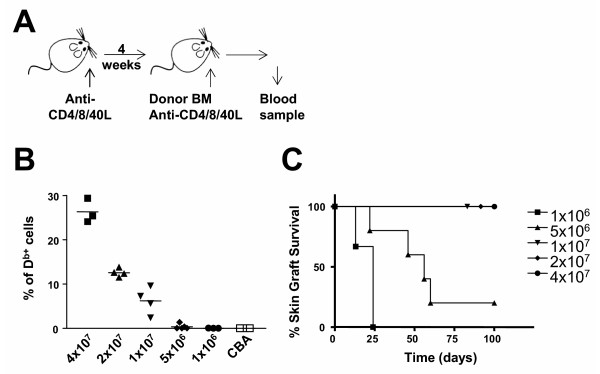
**Induction of BM engraftment with CD4, CD8 and CD40L mAbs**. (A) CBA mice were treated with three doses of 1 mg of non-depleting CD4, CD8 and CD40L mAbs on days -28, -26, -24, 0, 2 and 4 in relation to the day of BMT (day 0). (B) The level of hematopoietic chimerism was determined among peripheral blood mononuclear cells of CBA mice, transplanted with different numbers of B10 BM, by flow cytometry. CBA mice not subjected to BMT were used as a control group. Results are from day 120 following BMT. Difference from the control group is statistically significant in animals transplanted with 1×10^7 ^cells or more (*p *< 0.02). (C) The mice were transplanted with donor type (B10) skin grafts 50 days following BMT. Grafts survived indefinitely in animals where mixed chimerism had been established, being the difference between animals transplanted with 1×10^7 ^BM cells or more, and animals transplanted with 1×10^6 ^or 5×10^6 ^BM cells statistically significant (*p *< 0.02).

To establish the minimum number of marrow cells needed to achieve chimerism and tolerance, CBA mice were treated with three doses of 1 mg of each of the blocking mAbs to CD4, CD8 and CD40 L on alternate days 4 weeks prior to BMT, transplanted with different numbers of T cell-depleted B10 BM cells, and further treated with 1 mg of the same antibodies on day 0, 2 and 4 relative to BMT (Figure 1A). Peripheral blood samples were collected 50 days (not shown) and 120 days following BMT, and the level of chimerism was quantified by flow cytometry (Figure 1B). Chimerism was found to be stable since the levels detected at these two time points were always similar in any individual mouse. All animals transplanted with 1×10^7^, 2×10^7 ^or 4×10^7 ^T cell-depleted BM cells had detectable levels of chimerism, as did one mouse transplanted with 5×10^6 ^BM cells. All mice transplanted with 1×10^6 ^BM cells demonstrated no detectable chimerism.

To determine whether tolerance was dependent on the degree of chimerism, all mice were challenged with donor type skin grafts on day 50 following BMT. In addition, (BALB/c × B10)F_1 _skin was transplanted on day 120 to determine whether any tolerance observed was dominant, in which linked suppression [16] would be the expected outcome. All animals that had detectable chimerism accepted donor-type skin indefinitely (Figure 1C). (BALB/c × B10)F_1 _skin grafts were readily rejected by all mice (MST=13d) indicating that we could not elicit linked suppression and dominant tolerance.

These data demonstrate, therefore, that 1×10^7 ^T cell-depleted BM cells together with this particular antibody protocol, is sufficient to achieve stable mixed chimerism, and a non-dominant form of transplantation tolerance. Remarkably, this is achieved in the absence of myeloablative or myelosuppressive conditioning.

### Antibody first-stage treatment of the recipient is helpful in enabling bone marrow engraftment

**Figure 2:**
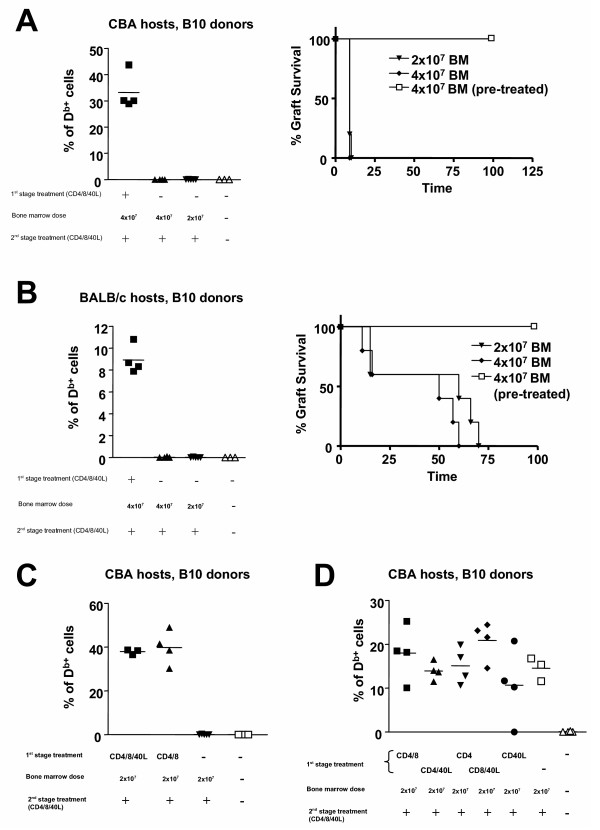
**The requirement for first-stage antibody treatmentin advance of BMT**. Except for control mice which received no treatment, all mice represented in this figure received CD4, CD8 and CD40L antibodies at the time of BMT (1 mg each ip on days 0, 2 and 4, relative to BMT on day 0). The first-stage antibody treatment was varied as described. CBA mice (A) and BALB/c mice (B) were transplanted with different doses of T cell depleted B10 BM, under the cover non-depleting CD4, CD8 and CD40L mAbs. Only one group of mice from each strain received the mAb treatment 4 weeks prior to BMT. Hematopoietic chimerism determined by flow cytometry 120 days following BMT is shown. In both strains the difference between untransplanted controls and animals not treated in advance of BMT is not statistically significant. Survival of B10 skin grafts, transplanted 50 days following BMT is represented. Only animals where mixed chimerism could be detected accepted the skin grafts indefinitely (*p *< 0.01 to any other group). (C) CBA mice were treated with 3 × 1 mg of CD4 and CD8 mAbs alone, or combined with the same dose of CD40L mAbs, 4 weeks before the transplantation of 4×10^7 ^T cell depleted B10 BM. One group was not treated at that time. Together with the BMT all animals were treated with CD4, CD8 and CD40L mAbs as described in Figure 1. Animals that did not receive BMT were used as negative controls. (D) Experiment identical to the one described in (C), using different combinations of mAb 4 weeks before BMT. No statistically significant difference was observed between the transplanted groups in the levels of hematopoietic chimerism 120 days following BMT.

We examined the requirements for a first-stage treatment in overcoming the immunologic resistance to BM engraftment. CBA or BALB/c mice were transplanted with different doses of donor BM and treated with 3 × 1 mg of CD4, CD8 and CD40L mAbs at the time of BMT, in the presence or absence of antibody treatment 4 weeks in advance of BMT. Only animals treated with the first-stage antibody-treatment in advance of BMT had detectable chimerism (Figure 2A and 2B). Mice transplanted with BM in the absence of first-stage treatment, readily rejected donor type skin grafts transplanted 50 days following BMT (Figure 2A and 2B).

The successful treatment regime used above was not, however, effective in B10 mice that had been transplanted with 2×10^7 ^T cell-depleted BM from CBA donors. Chimerism was less than 1% and all mice rejected donor-type skin (not shown).

Although antibody-treatment was critical at the time of BMT (as described below), first-stage treatment did not, however, seem to be an absolute requirement in all experiments. In one experiment, we observed that omission of the CD40L mAb made no difference to the efficacy of the first-stage treatment (Figure 2C), while in another we actually achieved long term engraftment in the absence of any first-stage treatment whatsoever (Figure 2D). Clearly, there are variations between experiments in the need for first-stage treatment, but more importantly, treatment with CD4 and CD8 mAbs does seem to guarantee routine success in permissive strains. It should be noted that CD8 mAbs on their own could not be tested for the first-stage treatment because they lead to sensitization to rat antibodies, nullifying the efficacy of subsequent antibody administration [17]. This does not happen when CD8 antibodies are combined with CD4 or CD40L antibodies.

To investigate the impact of the first-stage treatment on T cell populations, spleen cells from animals treated with CD4 and CD8 mAbs were collected 4 weeks following treatment (at the time donor BM is usually transplanted) and analysed by flow cytometry. Antibody-treated mice showed a marked reduction in CD8^+ ^T cells (Table 1). Relative to untreated controls, antibody-treated mice had a significantly higher frequency of CD44^+ ^cells amongst CD8^+ ^cells. Small but significant changes within the CD4^+ ^population were observed, including an increase of CD44^+ ^and CD25^+ ^cells in antibody-treated mice.

**Table 1:** T cell sub-populations in mAb treated mice. FACS analysis of splenocytes collected from untreated and antibody-treated CBA mice (1 mg YTS177 and 1 mg YTS105 ip given on days -28, -26 and -24 relative to data collection on day 0). 'Total splenocytes' refers to all cells in the lymphocyte forward- and side-scatter gate. NS (not significant) p > 0.05; (**) p < 0.001.

**Cell phenotype**	**Untreated, *n *= 6(mean +/- SD)%**	**AntiCD4 + antiCD8 treated, *n *6 (mean +/- SD)%**	***p *value**
(CD3^+^CD8^+^/total splenocytes)	20.8 +/- 2.6	1.8 +/- 0.6	**
^# ^(CD44^+ ^/CD8^+^)	6.9 +/- 0.8	36.7 +/- 6.4	**
(CD3^+^CD4^+ ^/total splenocytes)	32.0 +/- 6.2	37.8 +/- 6.2	NS
^# ^(CD25^+ ^/CD4^+^)	11.1 +/- 0.7	13.8 +/- 1.0	**
^# ^(CD44^+ ^/CD4^+^)	8.1 +/- 1.0	14.4 +/- 1.6	**
			

^# ^Only gated CD3^+ ^cells were taken into account for these calculations

### Anti-CD40L mAb is a necessary component of the second stage treatment

We investigated whether a 2-stage protocol consisting solely of co-receptor blockade with omission of co-stimulation blockade would still enable the engraftment of donor BM. All mice received the first-stage treatment consisting of co-receptor blockade alone initiated 4 weeks before transplantation. At the time of transfer of 2×10^7 ^T cell-depleted B10 bone marrow cells, the mice were split into three groups. One group received CD4, CD8 and CD40L mAbs; a second received CD4 and CD8 mAbs and a third received no mAbs. Chimerism was only achieved in the group which received the triple cocktail at the time of BM transplantation.

### Bone marrow engraftment in congenic mouse strains

Based on the failure of the 2-stage protocol to enable engraftment in B10 recipients of CBA bone marrow, we turned to a congenic system to provide a less stringent immunological barrier. In 2 experiments, no engraftment was observed in B6 recipients of congenic B6.CD45.1 bone marrow in the absence of antibody-treatment, while control CBA recipients which received the 2-stage protocol were chimeric (mean +/- SD = 6.4% +/- 1.5 and 4.3% +/- 1.3). B6 recipients of the 2-stage antibody protocol plus congenic B6.CD45.1 bone marrow showed low but significant levels of chimerism (mean +/- SD = 2.7% +/- 0.3 and 2.7% +/-0.6) (Figure 4A).

**Figure 3:**
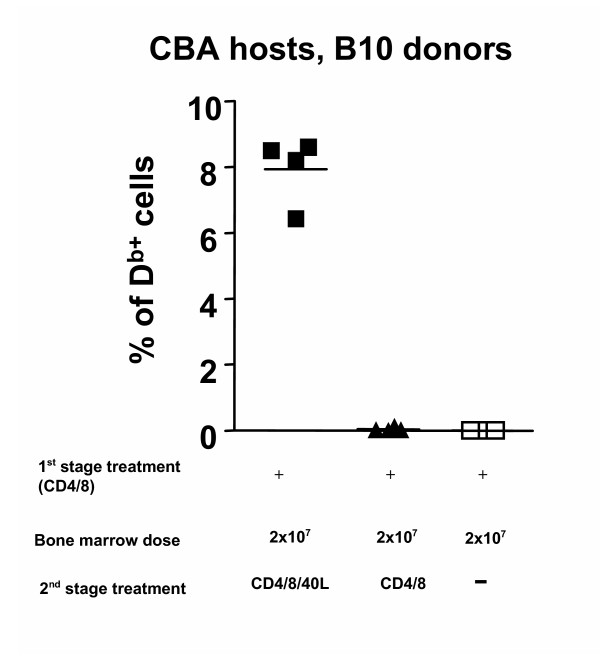
**MAb requirements at the time of BMT (second-stagetreatment)**. CBA mice were treated with non-depleting CD4 and CD8 mAbs alone 4 weeks before transplantation of 2 × 10^7 ^T cell depleted B10 BM. At the time of BMT the mice were treated with CD4 and CD8 mAb alone, or combined with CD40L mAb. Chimerism was only detected in the mice treated with both co-receptor and co-stimulation blockade at the time of BMT. Data is representative of 2 independent experiments.

**Figure 4:**
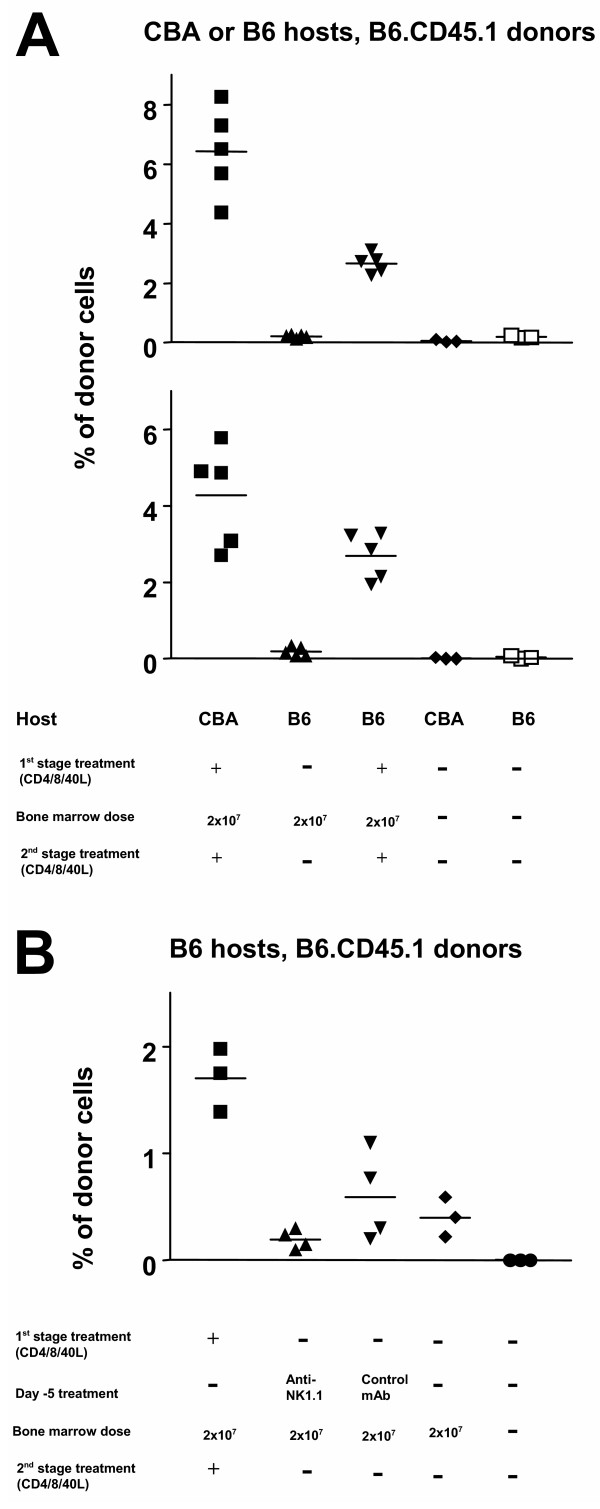
**BM engraftment in congenic mice**. A dose of 2×10^7 ^T cell depleted bone marrow cells from B6.CD45.1 mice was transplanted into CBA or congenic B6 mice treated with mAbs as described in figure 1. (A) One group of B6 mice was transplanted in the absence of any mAb treatment, and a group of CBA and B6 mice not subjected to BMT was used as a negative control. Hematopoietic chimerism was determined by quantification of peripheral blood mononuclear cells 120 days following BMT by flow cytometry. The results from two independent experiments are represented. The difference between the groups treated with mAb and any other group is statistically significant (*p *< 0.001). The difference between CBA and B6 recipients of B6.CD45.1 BM under the cover of mAbs is also significant in both experiments (*p *= 0.04 and *p *= 0.0006). (B) B6 mice were treated with 1 mg of the mAb PK136 administered 5 days prior to transplantation of B6.CD45.1 BM, to deplete their NK1.1 cells, while another group was treated with the control mAb YCATE55. Additional animals were subjected to the treatment described in Figure 1. NK cell depletion failed to achieve chimerism (*p *< 0.001).

We investigated whether BM engraftment in congenic mice might be facilitated by targeting NK cells. The specific targeting of NK cells by treatment with anti-NK1.1 antibody five days prior to BMT did not, however, enable BM engraftment (Figure 4B).

## Discussion

These new data show that a combination of antibodies targeting the CD4 and CD8 T cell co-receptors plus the CD40L molecule can facilitate bone marrow engraftment in the absence of any myelosuppressive conditioning of the recipient. A dose of marrow as low as 1×10^7 ^T cell-depleted BM (approximately 4×10^8 ^cells/kg) could establish mixed chimerism detectable 120 days following BMT. This state of mixed chimerism was sufficient to enable long-term acceptance of donor type skin allografts. Although the level of chimerism achieved was found to vary between experiments, we confirmed by titrating the dose of bone marrow, that even low levels of chimerism are maintained long-term, and these are compatible with long-term acceptance of skin grafts.

We have previously observed the resistance of B6 mice to tolerance induction to skin grafts by our antibody protocols [18]. In this study, the same was found for bone marrow transplants. A congenic system was employed to test the effect of the full 2-stage protocol in a system where the intensity of the allogeneic response is markedly reduced, although some antigenicity of the CD45.1 allele has been reported [19]. The levels of chimerism achieved in congenic B6 recipients were lower than fully allogeneic CBA recipients (Figure 4A), showing a relative resistance of B6 recipients to engraftment of donor hematopoeitic stem cells. The mechanism of resistance to engraftment is unclear, but it may be that 'resident' B6 stem cells enjoy an advantage in competing for 'niches', for instance by being more abundant than in the other strains [20]. There are several reports in which mixed chimerism was achieved in B6 recipients by combining co-stimulation blockade with either irradiation or 'megadoses' of bone marrow [11,12,21]. Interestingly, targeting CD40L alone was sufficient for chimerism in MHC-mismatched B10.BR recipients of B6 bone marrow [13], a donor-recipient combination which shares 'minors'. Unlike B6 mice, it seems B10.BR recipients (H-2^k ^with 'black minors') do not display resistance to donor stem cell engraftment, which therefore might be associated with the H-2^b ^haplotype of the MHC.

We were surprised by the variability in the requirement for the first-stage antibody-treatment in engraftment of donor stem cells. This variability between experiments may be explained by the combined treatment being capable of minimizing some component of heterologous immunity; that is, a heightened state of alloreactivity as a result of exposure to environmental antigens [22], which could vary between groups of experimental mice. The partial depletion of CD8^+ ^cells by the first-stage treatment (Table 1) was also unexpected because the isotype of the CD8 antibody used is rat IgG2a, which is the same isotype as the patently non-depleting CD4 antibody used. We have observed this same CD8 antibody to be non-depleting in previous studies [23]. This 'blocking' CD8 antibody may hinder TCR interactions with self MHC molecules, and the level of CD8^+ ^T cell loss might be determined, again, by environmental factors. Our data suggest when CD8^+ ^T cell loss occurred, it was predominantly seen in the antigen-inexperienced CD44^- ^pool, which could include alloantigen-specific naïve T cells. The first-stage treatment impacts the CD4^+ ^subset also, as there is a small but significant increase in the percentage of CD4^+ ^cells expressing memory and activation markers, such as CD44 and CD25. Taken together, our results indicate there are systematic variables surmounted reliably by the 2-stage protocol, which appears to ensure donor stem cell engraftment in susceptible strains.

The use of co-stimulation blockade with a CD40L mAb alone has been reported as effective in inducing BM engraftment, without the need for myeloblative conditioning, although this required a dose of BM one order of magnitude greater than in our present study [11,12]. Seung and collaborators have reported that BM engraftment (5×10^7 ^donor cells) can be facilitated, in the absence of myelosuppression, by prior infusion of DST under the cover of anti-CD40L mAbs, or co-administration of CD40L and CD122 antibodies in the absence of DST [13]. Furthermore, anti-CD40L mAbs have been also shown useful in enabling the engraftment of clinically attainable doses of bone marrow with reduced intensity conditioning regimes, even in resistant strains such as B6 mice [7-9]. Detailed mechanistic studies have identified a potent effect of CD40L antibodies on host T cells when allogeneic single cell suspensions are delivered intravenously (donor specific transfusion or bone marrow transplant). The propensity of host T cells, which recognize the alloantigens, to die under these circumstances is well described [24,25]. We report that anti-CD40L was an essential component of the treatment protocol at the time of transplantation to allow MHC-mismatched bone marrow to engraft. Further work has demonstrated that an aglycosyl CD40L mAb with impaired binding to complement and Fc receptors efficiently enabled engraftment in this protocol (Daley et al, manuscript in preparation).

## Conclusion

Previous studies showed that, in the absence of further conditioning, targeting either the T cell co-receptors [26] or the CD40L molecule [13]failed to enable BM engraftment if the mismatch was across both minor and major histocompatibility barriers. Our combined targeting of the CD4 and CD8 co-receptors and the CD40L molecule in a 2-stage protocol seems truly synergistic. Similar protocols offer an alternative to co-stimulation blockade alone in enabling the induction of mixed chimerism as part of a non-myeloablative protocol, or by allowing the reduction of the cytoreductive component, for treatment of non-malignant diseases, as well as for malignant diseases prior to the use of donor lymphocyte infusions to eradicate the tumour.

## Methods

### Mice

CBA/Ca (CBA, H-2^k^), BALB/c (H-2^d^), C57BL/10 (B10, H-2^b^), C57BL/6 (B6, H-2^b^) and B6.SJL.CD45 (B6.CD45.1, H-2^b^) mice were bred and maintained in specific pathogen free (SPF) facilities at the Sir William Dunn School of Pathology (Oxford, UK). The animals used in the experiments were sex-matched and between 8 and 10 weeks of age. Procedures were conducted in accordance with the Home Office Animals (Scientific Procedures) Act of 1986.

### Bone marrow transplantation

BM donors were depleted of T cells with an i.p. injection of 1 mg of the anti-CD8 mAbs YTS156 and YTS169, and the anti-CD4 mAbs YTS191 and YTA3.1 five days prior to BM collection [27]. BM was collected by flushing the femurs and tibias with RPMI medium. The cells were counted, resuspended in PBS and injected intravenously into recipient mice.

### Skin grafting

Mice were anaesthetised with a mixture of 1 mg/ml Xylazine (Rompun^®^, Bayer) and 10 mg/ml Ketamine (Ketaset^®^, Fort Dodge). 0.2 ml per 20 g of body weight was injected i.p. Skin grafting was performed as described elsewhere [28]. Briefly, skin grafting was conducted by grafting full thickness tail-skin (1 × 1 cm) on the lateral flank. Grafts were observed on alternate days after the removal of the bandage at day 7 and considered rejected when no viable donor skin was present.

### Treatment with mAbs

BMT were performed under the cover of 1 mg YTS177.9, 1 mg YTS105.18 and 1 mg MR1 [29] as described in the text [15]. All mAbs were produced in our laboratory by culture in hollow fibre bioreactors, purified from culture supernatants by 50% ammonium sulphate precipitation, dialysed against PBS, and the purity checked by native and SDS gel electrophoresis (PhastGel, Pharmacia, St. Albans, UK).

### Flow cytometry

Hematopoietic chimerism was quantified by staining peripheral blood with mAbs specific for H-2D^b^, H-2K^k ^and H-2K^d ^(all from BD Biosciences). To distinguish cells from the congenic strains we used mAbs specific for CD45.1 and CD45.2 (BD Biosciences). To study the effect of first-stage antibody treatment only, splenocytes were subjected to osmotic lysis of red blood cells before staining with mAbs specific for CD3, CD4, CD8, CD25 and CD44 (all from BD Biosciences). Cells were analysed with a FACScalibur (BD Biosciences) and CellQuest software (BD Biosciences).

### Statistical analysis

Statistical analysis of graft survival was made by the log rank method. P values in Table 1 were calculated by the student's T test (unpaired, 2-tailed).

## Authors' contributions

LG participated in the study design, performed research, analysed data, and drafted the manuscript. SD performed research, analysed data, and contributed to the manuscript. PJF performed research. SPC participated in the study design and data analysis. HW participated in the study design, data analysis, and manuscript preparation. All authors read and approved the final manuscript.

## References

[B1] Buckley RH (2004). A historical review of bone marrow transplantation for immunodeficiencies. J Allergy Clin Immunol.

[B2] Krivit W, Aubourg P, Shapiro E, Peters C (1999). Bone marrow transplantation for globoid cell leukodystrophy, adrenoleukodystrophy, metachromatic leukodystrophy, and Hurler syndrome. Curr Opin Hematol.

[B3] Openshaw H, Nash RA, McSweeney PA (2002). High-dose immunosuppression and hematopoietic stem cell transplantation in autoimmune disease: clinical review. Biol Blood Marrow Transplant.

[B4] Messner RP (1997). The potential of bone marrow stem cell transplantation in the treatment of autoimmune diseases. J Rheumatol.

[B5] Wekerle T, Sykes M (2001). Mixed chimerism and transplantation tolerance. Annu Rev Med.

[B6] Sykes M, Sachs DH (2001). Mixed chimerism. Philos Trans R Soc Lond B Biol Sci.

[B7] Adams AB, Durham MM, Kean L, Shirasugi N, Ha J, Williams MA, Rees PA, Cheung MC, Mittelstaedt S, Bingaman AW, Archer DR, Waller EK, Larsen CP (2001). Costimulation blockade, busulfan, and bone marrow promote titratable macrochimerism, induce transplantation tolerance, and correct genetic hemoglobinopathies with minimal myelosuppression. J Immunol.

[B8] Takeuchi Y, Ito H, Kurtz J, Wekerle T, Ho L, Sykes M (2004). Earlier low-dose TBI or DST overcomes CD8+ T-cell-mediated alloresistance to allogeneic marrow in recipients of anti-CD40L. Am J Transplant.

[B9] Taylor PA, Lees CJ, Waldmann H, Noelle RJ, Blazar BR (2001). Requirements for the promotion of allogeneic engraftment by anti-CD154 (anti-CD40L) monoclonal antibody under nonmyeloablative conditions. Blood.

[B10] Bachar-Lustig E, Rachamim N, Li HW, Lan F, Reisner Y (1995). Megadose of T cell-depleted bone marrow overcomes MHC barriers in sublethally irradiated mice. Nat Med.

[B11] Durham MM, Bingaman AW, Adams AB, Ha J, Waitze SY, Pearson TC, Larsen CP (2000). Cutting edge: administration of anti-CD40 ligand and donor bone marrow leads to hemopoietic chimerism and donor-specific tolerance without cytoreductive conditioning. J Immunol.

[B12] Wekerle T, Kurtz J, Ito H, Ronquillo JV, Dong V, Zhao G, Shaffer J, Sayegh MH, Sykes M (2000). Allogeneic bone marrow transplantation with co-stimulatory blockade induces macrochimerism and tolerance without cytoreductive host treatment. Nat Med.

[B13] Seung E, Mordes JP, Rossini AA, Greiner DL (2003). Hematopoietic chimerism and central tolerance created by peripheral-tolerance induction without myeloablative conditioning. J Clin Invest.

[B14] Ildstad ST, Chilton PM, Xu H, Domenick MA, Ray MB (2004). Preconditioning of NOD mice with anti-CD8 mAb and co-stimulatory blockade enhances chimerism and tolerance and prevents diabetes while depletion of {alpha}{beta}-TCR+ and CD4+ cells negates the effect. Blood.

[B15] Graca L, Le Moine A, Lin CY, Fairchild PJ, Cobbold SP, Waldmann H (2004). Donor-specific transplantation tolerance: the paradoxical behavior of CD4+CD25+ T cells. Proc Natl Acad Sci U S A.

[B16] Davies JD, Leong LY, Mellor A, Cobbold SP, Waldmann H (1996). T cell suppression in transplantation tolerance through linked recognition. J Immunol.

[B17] Benjamin RJ, Cobbold SP, Clark MR, Waldmann H (1986). Tolerance to rat monoclonal antibodies. Implications for serotherapy. J Exp Med.

[B18] Davies JD, Cobbold SP, Waldmann H (1997). Strain variation in susceptibility to monoclonal antibody-induced transplantation tolerance. Transplantation.

[B19] van OS R, Sheridan TM, Robinson S, Drukteinis D, Ferrara JL, Mauch PM (2001). Immunogenicity of Ly5 (CD45)-antigens hampers long-term engraftment following minimal conditioning in a murine bone marrow transplantation model. Stem Cells.

[B20] de Haan G, Nijhof W, Van Zant G (1997). Mouse strain-dependent changes in frequency and proliferation of hematopoietic stem cells during aging: correlation between lifespan and cycling activity. Blood.

[B21] Wekerle T, Sayegh MH, Hill J, Zhao Y, Chandraker A, Swenson KG, Zhao G, Sykes M (1998). Extrathymic T cell deletion and allogeneic stem cell engraftment induced with costimulatory blockade is followed by central T cell tolerance. J Exp Med.

[B22] Adams AB, Williams MA, Jones TR, Shirasugi N, Durham MM, Kaech SM, Wherry EJ, Onami T, Lanier JG, Kokko KE, Pearson TC, Ahmed R, Larsen CP (2003). Heterologous immunity provides a potent barrier to transplantation tolerance. J Clin Invest.

[B23] Scully R, Qin S, Cobbold S, Waldmann H (1994). Mechanisms in CD4 antibody-mediated transplantation tolerance: kinetics of induction, antigen dependency and role of regulatory T cells. Eur J Immunol.

[B24] Iwakoshi NN, Mordes JP, Markees TG, Phillips NE, Rossini AA, Greiner DL (2000). Treatment of allograft recipients with donor-specific transfusion and anti-CD154 antibody leads to deletion of alloreactive CD8+ T cells and prolonged graft survival in a CTLA4-dependent manner. J Immunol.

[B25] Quezada SA, Fuller B, Jarvinen LZ, Gonzalez M, Blazar BR, Rudensky AY, Strom TB, Noelle RJ (2003). Mechanisms of donor-specific transfusion tolerance: preemptive induction of clonal T-cell exhaustion via indirect presentation. Blood.

[B26] Leong LY, Qin S, Cobbold SP, Waldmann H (1992). Classical transplantation tolerance in the adult: the interaction between myeloablation and immunosuppression. Eur J Immunol.

[B27] Cobbold SP, Martin G, Waldmann H (1990). The induction of skin graft tolerance in major histocompatibility complex-mismatched or primed recipients: primed T cells can be tolerized in the periphery with anti-CD4 and anti-CD8 antibodies. Eur J Immunol.

[B28] Graca L, Thompson S, Lin C-Y, Adams E, Cobbold SP, Waldmann H (2002). Both CD4+CD25+ and CD4+CD25- regulatory cells mediate dominant transplantation tolerance. J Immunol.

[B29] Noelle RJ, Roy M, Shepherd DM, Stamenkovic I, Ledbetter JA, Aruffo A (1992). A 39-kDa protein on activated helper T cells binds CD40 and transduces the signal for cognate activation of B cells. Proc Natl Acad Sci U S A.

